# Impact of Nanoparticles on Brain Health: An Up to Date Overview

**DOI:** 10.3390/jcm7120490

**Published:** 2018-11-27

**Authors:** Daniel Mihai Teleanu, Cristina Chircov, Alexandru Mihai Grumezescu, Adrian Volceanov, Raluca Ioana Teleanu

**Affiliations:** 1 Emergency University Hospital, Bucharest, Romania, “Carol Davila” University of Medicine and Pharmacy, 050474 Bucharest, Romania; telepapa@hotmail.com (D.M.T.); raluca.teleanu@umfcd.ro (R.I.T.); 2 Faculty of Engineering in Foreign Languages, 060042 Bucharest, Romania; cristina.chircov@yahoo.com; 3 Department of Science and Engineering of Oxide Materials and Nanomaterials, Faculty of Applied Chemistry and Materials Science, Politehnica University of Bucharest, 011061 Bucharest, Romania; grumezescu@yahoo.com; 4 ICUB—Research Institute of University of Bucharest, University of Bucharest, 36-46 M. Kogalniceanu Blvd., 050107 Bucharest, Romania

**Keywords:** nanoparticles, zero-dimensional, organic nanoparticles, inorganic nanoparticles, diagnosis, treatment, brain diseases, brain tumors, neurodegenerative disorders, brain health

## Abstract

Nanoparticles are zero-dimensional nanomaterials and, based on their nature, they can be categorized into organic, inorganic, and composites nanoparticles. Due to their unique physical and chemical properties, nanoparticles are extensively used in a variety of fields, including medicine, pharmaceutics, and food industry. Although they have the potential to improve the diagnosis and treatment of brain diseases, it is fundamentally important to develop standardized toxicological studies, which can prevent the induction of neurotoxic effects. The focus of this review is to emphasize both the beneficial and negative effects of nanoparticles on brain health.

## 1. Introduction

Expanding the use of nanomaterials in the fields of biomaterials, biosensors, nanoelectronics, or catalysis is of great interest due to their unique and innovative properties. These properties stem from their reduced size, since nanomaterials are defined as materials with at least one dimension less than 100 nanometers [1]. Based on their structure, nanomaterials can be categorized into zero-dimensional, including nanoparticles and quantum dots, one-dimensional, including nanofibers, nanotubes, and nanowires, two-dimensional, including graphene and graphene oxide [2,3], and three-dimensional nanomaterials, generally known as bulk nanomaterials, which consist of equiaxed nanometer sized grains, and they are characterized by three arbitrary dimensions which are not confined to the nanoscale [4]. Exceptionally, some materials could be placed at the border of these categories. Moreover, nanomaterials can either occur naturally or be chemically, physically, biologically, or mechanically synthesized [2].

**Figure 1:**
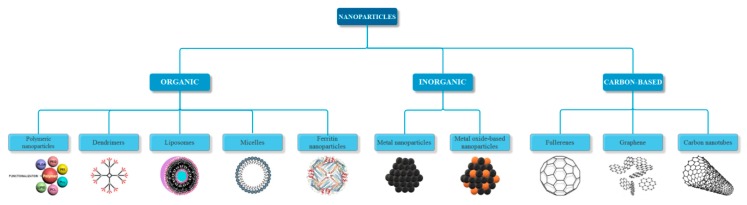
Classification of nanoparticles based on their nature.

As the subject of this review, nanoparticles have been defined by the International Organization for Standardization (ISO) as nano-objects with all three external dimensions in the nanoscale. Additionally, the terms nanorod or nanoplate could be used if the lengths of the longest and the shortest axes differ significantly, usually by more than three times [4]. Nanoparticles can be obtained in different sizes and shapes, such as spheres, cylinders, cubes, triangles, rings, or disks with various dimensions [5,6]. Furthermore, the generally accepted classification of nanoparticles is based on their nature, namely organic, inorganic, and carbon-based (Figure 1). The organic nanoparticles, also known as polymeric nanoparticles, are typically biodegradable and non-toxic, and they include dendrimers, ferritin nanoparticles, and hallow spheres, such as micelles and liposomes. The inorganic nanoparticles are mainly metal-based, such as silver, gold, iron, copper, aluminum, cadmium, cobalt, and zinc, and metal oxide-based nanoparticles, including titanium oxide, iron oxide, magnetite, silicon dioxide, cerium oxide, and zinc oxide, which possess improved properties when compared to their metal counterparts [7,8]. The carbon-based nanoparticles consist entirely of carbon, and they can be classified into fullerenes, graphene, and carbon nanotubes [9,10]. Since nanoparticles are the link between bulk materials and their molecular or atomic structure, their unique properties mostly depend on discreet molecular and atomic phenomena. Hence, their physical, chemical, and biological properties differ significantly when compared to particles at higher scales. The most important properties, regardless of their nature, which led to the intensive use of nanoparticles are: increased surface area, optical properties, such as quantum effects and increased absorption efficiency, uniformity, functionalization, quantum confinement, a phenomenon that causes spontaneous properties of semiconductivity, conductivity or electric insulation for adjacent particles [9,11], increased reactivity or stability in chemical processes, and enhanced mechanical and magnetic properties [9].

There has been a growing interest over the last years in using nanoparticles for a variety of biomedical applications. Thus, the most important applications regard the diagnosis and treatment of different diseases, through bioimaging, biosensors, targeted drug delivery, hyperthermia, and photoablation therapy [12], but also the targeted delivery of vaccines and genes. As a future perspective, theranostic nanoparticles, which are characterized by the capacity to simultaneously diagnose and treat, are being developed [13]. Similarly, nanoparticles have been utilized for imaging and treating brain diseases, including brain cancer and central nervous system disorders, which are poorly treated widespread diseases [14].

However, the use of nanoparticles for these applications may induce toxicity to the organism, especially due to their specific properties, such as the increased surface area which results in increased reactivity and biological activity. Thus, the contact with nanoparticles might cause permanent damage to the central nervous system. Although a high dosage is not necessarily correlated to an increased toxicity, understanding the nanoparticles biokinetics is essential to continuously use them in biomedical applications [15].

## 2. Routes of Exposure

The exposure-dose-response relationship is the basic principle of toxicology. Thus, it can be assumed that if nanoparticles enter the body, toxicological responses might be expected [16]. Although there is an extensive use of nanoparticles in biomedical and industrial applications, the toxicological effects on the health of living organisms are not completely known. The main routes of exposure to nanoparticles are through ingestion, inhalation, skin absorption, and injection. After entering the body, nanoparticles can reach the organs through systemic circulation. Furthermore, depending on their characteristics, such as size, shape, and chemical reactivity, they can cross the blood-brain barrier, or they can reach the brain through axonal transport along the olfactory nerve [17]. Since the exposure might also be unintentional, knowledge of the potential toxicities of these nanoparticles on the different organ systems and the correlation between the exposure route and its effects is crucial [18].

### 2.1. Exposure through Ingestion

In the recent years, nanoparticles have been intensively used in the food industry for the development of new tastes and texture features, the improvement of nutrient absorption capacity and food products packaging, and the synthesis of food ingredients, supplements, and additives [19]. Additionally, nanoparticles could be found in some unintended, nonedible products, such as food and drink containers and silver nanoparticles-coated toothbrushes [16]. Despite their beneficial effects, there are some safety concerns that should be addressed [19]. Furthermore, the oral administration of drug encapsulated nanoparticles, which offer improved bioavailability through various mechanisms, is the preferred route due to its simplicity [20].

After ingestion, nanoparticles encounter a series of barriers, including the gastric and intestinal milieu, the mucus barrier, the tight junctions blocking paracellular passage, the epithelial cells of the gastrointestinal tract, and the subepithelial tissue [21]. Subsequently, there are two pathways for nanoparticles absorption, specifically the paracellular absorption, which occurs when the nanoparticles are small enough to pass through the spaces between the cells, and the transcellular uptake through enterocytes, either passively or by binding to specific receptors [22]. Hence, depending on the size, dispersibility, and the charge of the nanoparticle [16], they can diffuse through the gastrointestinal tract into the systemic circulation and translocate to other organs [23]. If the size is reduced, nanoparticles can cross the blood-brain barrier and accumulate in the brain [24].

### 2.2. Exposure through Inhalation

In terms of accidental inhalation, the nanoparticles that pose a high risk are those found in the form of powders, suspensions, or sprays, which are ubiquitously used in textiles, paints, cosmetics, water disinfectants, and food packaging [25]. Depending on the size, there are several uptake possibilities: larger particles, with a diameter in the range of 5–30 µm, tend to remain in the nasopharyngeal region, particles in the range of 1–5 µm accumulate in the tracheobronchial region, and smaller particles, with a diameter between 0.1 and 1 µm will reach the alveolar region. Alternatively, nanoparticles with dimensions lower than 0.5 µm can cross the thin epithelium and reach the blood capillaries [26]. Furthermore, nanoparticles found in the nasal cavity can either cross the respiratory epithelium and reach the underlying blood vessels, or they can be absorbed through the olfactory epithelium and reach the brain [27].

However, the toxicological studies of inhaled nanoparticles are mostly performed on non-therapeutic types, which often show morbidity and mortality. These types of nanoparticles differ from the therapeutic nanoparticles, since the former are characterized by considerably reduced sizes, inorganic nature, water insolubility, and different dose and dosing frequency. Thus, the toxicological reports cannot be translated to nanoparticles that are used for biomedical applications [28].

### 2.3. Exposure through Skin Contact

The skin is a lipophilic medium that has the capacity to absorb both lipophilic and hydrophilic molecules through different routes. Depending on their physicochemical properties, nanoparticles can permeate the skin through various pathways that facilitate the entry and transport to the systemic circulation [29]. The main absorption routes of small nanoparticles are intracellularly through corneocytes, intercellularly around corneocytes, or via dermal structures like hair follicles and sweat glands [19,30].

For nanoparticles that are used in biomedical applications, the size and the ionizing potential are key aspects in the bioavailability of the substance, and the integrity of the skin will influence the absorption. However, toxicological studies show that both penetration and permeation of nanoparticles through the skin are limited to certain types [31].

### 2.4. Exposure through Injection

The exposure to nanoparticles through intravenous or intramuscular injection occurs intentionally and almost exclusively in nanomedicine [16]. The main purposes of their application are the diagnosis and therapeutics of various diseases. Some of the directions that concern this review are the applications regarding the diagnosis and treatment of brain cancer and central nervous system disorders, which require nanoparticles that are capable of crossing the blood brain barrier.

## 3. The Use of Nanoparticles for the Diagnosis and Treatment of Brain Diseases

Comprised of hundreds of various highly organized subtypes of neurons and glia, the central nervous system is the most complex and specialized body system. Similarly, the central nervous system disorders are equally complex, with each causing a collection of diagnostically definitive disruptions in behavior [32]. Consequently, the strategies for the treatment of these disorders are insufficient due to the existence of the blood brain barrier, which is comprised of various cell types, cellular interfaces with tight junctions, extracellular matrix components, and transporter mechanisms. The blood brain barrier is a selective permeability system that is responsible for the protection of the brain tissue against the exposure to foreign substances through the blood or the cerebrospinal fluid [33]. Hence, a limited amount of drug enters the central nervous system, which is insufficient for the treatment of brain diseases. Therefore, novel strategies for the delivery of therapeutic agents to the brain, such as nanotechnology approaches, are necessary for an effective treatment of central nervous system disorders [34]. The use of nanoparticles to diagnose brain diseases or to assist the delivery of drugs across the blood brain barrier has gained a great interest, since they offer a series of advantages, namely targeting efficiency, non-invasiveness, biodegradability, stability, and controllability to load and release drugs [35].

### 3.1. Nanoparticle Applications for Brain Tumors

The development of novel applications for brain tumors diagnosis and treatment has received the attention from scientists due to their high prevalence. Intracranial metastases from systemic cancers, meningiomas, and gliomas are the most common and they require complex multidisciplinary care, including neurosurgery, radiation oncology, and medical oncology [36].

The use of different types of nanoparticles for the detection of brain tumors through various bioimaging techniques has been reported. Hence, studies report the use of iron oxide nanoparticles functionalized with phosphonate polyethylene glycol and covalently coupled to the cyclo RGD peptide sequence as magnetic resonance imaging contrast agents due to their magnetic properties [37]. Furthermore, by coating the iron oxide nanoparticles with bovine serum albumin, conjugating the tumor-specific ligand folic acid onto them, and labeling with fluorescein isothiocyanate, an enhanced intracellular dual-modal imaging, specifically the magnetic resonance imaging and intracellular visualization, for brain tumors can be obtained [38]. Alternatively, multilayered semiconducting polymer nanoparticles containing a hydrophobic semiconducting polymer that was coated with an optically inner silica shell have been synthetized for brain tumors imaging using both fluorescence and photoacoustic brightness [39].

Drug encapsulated nanoparticles are usually polymeric, especially due to their favorable biodegradability. Studies reported the use of liposomes as vehicles for the delivery of anti-cancer drugs, including doxorubicin and erlotinib [40,41]. The results showed a considerably higher translocation across the blood brain barrier, both for in vitro brain tumor models [40] and for numerical simulations using a three-dimensional brain tumor model reconstructed from magnetic resonance images [41]. Moreover, poly(lactic-co-glycolic acid) nanoparticles [42], block copolymer nanoparticles consisting of polyethylene glycol and poly(ω-pentadecalactone-co-p-dioxanone) [43], or of polyethylene glycol and poly(lactic-co-glycolic acid) [44], and hybrid nanoparticles using poly(lactic-co-glycolic acid), 1,2-distearoyl-sn-glycero-3-phosphoethanolamine-N-(carboxy-poly(ethylene glycol)), and charged 1,2-dioleoyl-3-trimethylammonium-propane [45] have been used for the delivery of various anti-cancer drugs, all showing improved targeting and drug release efficiency, with a decrease in brain tumor size.

A novel approach in brain cancer therapy is the use of theranostic nanoparticles, which have the capacity to simultaneously image and treat specific brain tumors. Imaging is usually performed through near-infrared fluorescence light by modifying the polymeric substrate of the nanoparticles with photosensitizer agents [46,47]. The treatment of the tumors is accomplished by the encapsulation of therapeutic agents [47] or by photothermal therapy, which uses the photothermal conversion agents to generate heat for cancer cell ablation when near-infrared laser irradiation occurs [46,48].

### 3.2. Nanoparticle Applications for Neurodegenerative Disorders

Neurodegenerative diseases are age-dependent disorders that pose a great concern for human health due to the increasing prevalence. The most common neurodegenerative diseases are Alzheimer’s disease and Parkinson’s disease [49], and their pathophysiology is diverse, including memory impairments, cognitive defects, locomotor dysfunction, and emotional and behavioral problems [49,50]. The main mechanism for these diseases is the slow and progressive neuronal dysfunction, which causes the loss of neurons in the central nervous system, leading to functional loss or sensory dysfunction [50].

Furthermore, neurodegenerative diseases are characterized by the conformational change of native proteins, which results in the aggregation and formation of insoluble amyloid fibrils. Thus, by developing adequate tools that can detect these formations, the diagnosis and treatment of these diseases might be improved [51]. The use of nanoparticles for amyloid fibrils detection mainly focuses on inorganic materials, such as magnetic nanoparticles, including magnetite nanoparticles [52] and gadolinium-based nanoparticles [53] and plasmonic nanoparticles [51].

Moreover, intravenous administered nanoparticles are promising delivery systems for the functional recovery in neurodegenerative pathologies [54]. In the case of Alzheimer’s disease, which is a form of dementia resulting in issues regarding memory, cognition, and behavior [55], biodegradable polymeric nanoparticles consisting of polyethylene glycol and/or poly(lactic-co-glycolic acid) and functionalized with specific antibodies [56,57] or oligopeptide drugs [58] have been used to eliminate and prevent the formation of amyloid fibrils, leading to the disease. For the treatment of Parkinson’s disease, which is associated with motor features, such as rest tremor, bradykinesia, rigidity and postural instability, olfactory dysfunction, cognitive impairment, psychiatric symptoms, and autonomic dysfunction [59], various types of nanoparticles have been studied. Some of these studies reported ex vivo of chitosan nanoparticles for the delivery of Selegiline, a well-known anti-Parkinson agent [60], and Pramipexole [61], a non-ergot based dopamine that effectively counters Parkinson’s diseases progression [62]. Additionally, the use of cerium oxide nanoparticles, which protect neurons against reactive oxygen species-induced damage, has resulted in the improvement of motor dysfunctions and decreased apoptosis [63]. Another approach for the treatment of Parkinson’s disease is to use polymeric nanoparticles that can efficiently deliver microRNA, which might induce migration of neurons into the lesioned site and ameliorate motor symptoms [64].

### 3.3. Nanoparticle Applications for Stroke

Stroke represents a major medical emergency that can lead to disability or death. There are two types of stroke, namely the ischemic stroke, which occurs when a cerebral blood vessel is blocked, and the hemorrhagic stroke, which is caused by the rupture of the cerebral blood vessel. The more prevalent type is the ischemic stroke, which results in neurological death, inflammation, and damage to the neurovascular unit, leading to severe neurological symptoms [65]. Unfortunately, there are limited available treatments for ischemic stroke, and the development of strategies for both immediate treatment and post-stroke recovery is crucial [66]. Presently, there are studies that are focused on the use of inorganic and organic nanoparticles, including polymeric nanoparticles, liposomes, and metal and metal oxides nanoparticles, for stroke therapy [67,68].

One study reported the synthesis of poly(lactic-co-glycolic acid) nanoparticles, functionalized with chlorotoxin as a target ligand and encapsulating Lexiscan, which can enhance the blood brain barrier permeability, and Nogo-66, a highly effective receptor antagonist peptide in strokes. Results showed improved stroke survival, thus proving the potential of this system for stroke therapy [69]. Another study used superparamagnetic iron oxide nanoparticles for the delivery of siRNA and tracking of endothelial progenitor cells, which have been studied due to their potential in ischemic stroke therapy. Additionally, the use of hif-prolyl hydroxylase 2 silencing may improve the efficacy of the system through increased migration and survival ability of the cells [70].

## 4. Harmful Effects of Nanoparticles on Brain Health

The field that focuses on the study of the adverse effects that are caused by the exposure to nanomaterials is toxicology, or, more precisely, nanotoxicology [71]. As an aspect of nanoscience, the physicochemical characterization of these nanomaterials with respect to several factors that regard their specific properties and interactions with living tissues is necessary [72,73]. Nanotoxicology is rapidly growing especially due to the increasing applications of nanomaterials in biomedicine, biotechnology, and environmental industry and the development of standardized nanotoxicological studies is crucial [74,75,76]. Nanotoxicology is also necessary since the interactions of nanoparticles with living cells and tissues are often unpredictable due to their unique physicochemical properties [77,78]. Additionally, considering the fact that the dimensions of synthetic nanoparticles is close to the dimensions of the cells, their action might interfere with vital cellular processes [79,80,81]. Unfortunately, the physicochemical characteristics that are responsible for the unique properties of nanoparticles that account for their beneficial effects in medical applications, also lead to their toxicity, resulting in human and environmental health concerns [72,77]. Moreover, the unusual toxic effects of nanoparticles might be considerably different than those of the bulk materials [81]. As such, understanding these health concerns is fundamentally necessary for the successful application of nanoparticles in biomedicine [82].

**Figure 2:**
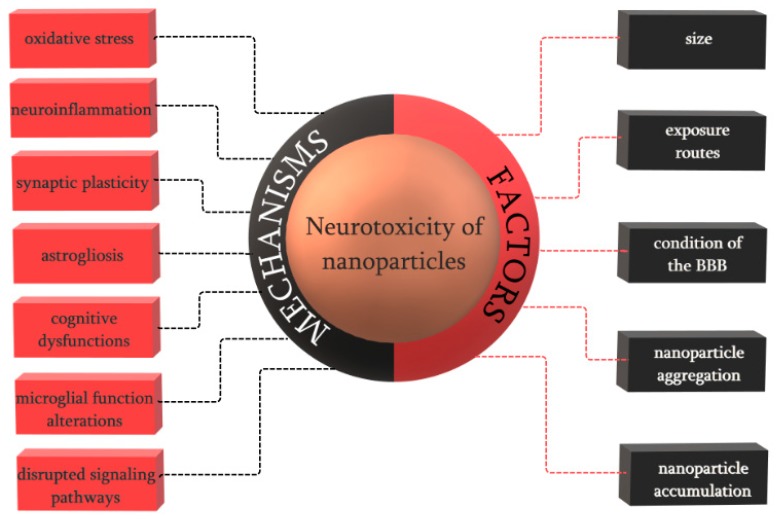
The main mechanisms and factors of neurotoxicity induced by nanoparticles.

Through any route of exposure, after crossing the blood brain barrier, nanoparticles have a tendency to accumulate in specific brain regions, where they can access the neural cells, including neurons, astrocytes, and microglia [24]. The field that studies the functional and structural changes of the nervous system that are caused by the exposure to foreign substances is neurotoxicology. Thus, neurotoxicity is defined as any adverse effect on the structure, function, or chemistry of the central nervous system as a consequence of biological, chemical, or physical influences (Figure 2). The neurotoxic effect can either result in the direct alteration of the structure or activity of the neural system or it can lead to subsequent effects due to glial activations and glial-neuronal interactions [83]. Furthermore, neurotoxicity can manifest through various mechanisms, including oxidative stress, resulting in cell apoptosis and autophagy, immune responses, and neuroinflammation, which will affect the blood brain barrier function [84].

The neurotoxicity of nanoparticles is usually caused by the extensive production of reactive oxygen species that lead to oxidative stress (Table 1). Subsequently, the release of cytokines causes neuroinflammation, and finally, through apoptotic mechanisms, neuronal death [85]. Many of the commonly used nanoparticles, including titanium dioxide nanoparticles, iron oxide nanoparticles, silver nanoparticles, gold nanoparticles, silica nanoparticles, and carbon-based nanoparticles, have been reported as potentially neurotoxic materials [86].

The administration of titanium oxide nanoparticles through any route leads to the absorption and translocation into the brain, which can affect brain development and function. Furthermore, they can cross the placental barrier and accumulate in the fetal brain, causing impairments in the fetal brain development [87]. The main mechanisms of neurotoxicity induced by titanium oxide nanoparticles are the oxidative stress, inflammatory responses, apoptosis, genotoxicity, and impairment of cell components [88]. Additionally, neurotoxicity may occur through dysregulated neurotransmitters, disturbed distribution of trace elements, synaptic plasticity, and disrupted signaling pathways [89].

The ability of iron oxide nanoparticles to reach the brain by crossing the blood brain barrier or through the olfactory nerve has gained the interest for their application in drug delivery and imaging diagnostics in the nervous system. However, this ability is also responsible for their neurotoxicity, since studies reported that daily exposure to iron oxide nanoparticles affects synaptic transmissions and nerve conduction, causing neural inflammation, apoptosis, induced neural antioxidant responses, and immune cell infiltration [90].

Since silver nanoparticles are increasingly used in biomedical applications and daily use consumer products, the study of their neurotoxic effects, mostly on neurotransmitters, is fundamental [91]. The mechanisms of neurotoxicity induced by silver nanoparticles are specific to most nanoparticles, including the induction of oxidative stress, mitochondrial damage, and an increase in the calcium levels related to transporter/receptor mechanisms. Moreover, neurotoxicity might be influenced by their size, shape, surface coatings, rates of silver ions release, and interactions with specific cells and proteins [92].

There are two main routes for the neuronal uptake of gold nanoparticles, namely through the olfactory nerves and by crossing the blood brain barrier. In the latter case, gold nanoparticles might cause three types of severe neurological pathologies: astrogliosis, also known as reactive astrocytosis, characterized by an increased number of astrocytes that is caused by the death of adjacent neurons, which leads to scar formation and the inhibition of axon regeneration, increased seizure activity, defined as any change in the electrical activity of the brain, and cognition defects, such as attention, memory, and judgement impairments [93].

Exposure to silica nanoparticles results in neurotoxic effects, whereas very low levels increase the oxidative stress and alter the microglial function, with a highly negative impact on the striatum and dopaminergic neurons [94]. While previous studies have demonstrated the neurotoxicity of silica nanoparticles, recent work has been focusing on their role in neurodegeneration. The intranasal administration of silica nanoparticles leads to cognitive dysfunction and impairment, but also to pathologies that are similar to neurodegeneration and synaptic changes [95]. Furthermore, the impact on the electrical activity produced by changes in the membrane potential leading to potential genotoxicity after exposure to silica nanoparticles has been studied. Results showed that despite the induction of depolarization in the neuronal membrane potential, the functional behavior was not affected and there were no changes in gene expression detected [96].

The application of carbon-based nanoparticles faces a major challenge in terms of toxicity on neuronal cells. Studies showed that the inhalation of carbon nanoparticles leads to the accumulation in the olfactory bulb, inducing an inflammatory response by activating the microglial cells. Their neurotoxicity may be correlated to different diameters and/or lengths. However, there are ways to limit the neurotoxicity of carbon nanoparticles by functionalization with hydrophilic polymers, thus improving their water solubility and dispersion, or by shortening their lengths [97].

## 5. Conclusions and Perspectives

Since the application of nanoparticles, defined as nano-objects with all three external dimensions in the nanoscale, in the diagnosis and treatment of brain diseases, including brain tumors, neurodegenerative disorders, and stroke, is continuously and rapidly emerging, the need for understanding both their beneficial and negative impacts on brain health is imperative. Although nanoparticles possess unique physicochemical properties that justify their broad use in applications for the central nervous system, they can also manifest neurotoxic effects, including oxidative stress, resulting in cell apoptosis and autophagy, immune responses, and neuroinflammation, which will affect the blood brain barrier function. Thus, the development of standardized toxicological studies is crucial for the improvement of brain applications.

## Figures and Tables

**Table 1:** Biomedical applications and the related neurotoxic effects of different nanoparticles.

Nanoparticle	Biomedical Applications	Neurotoxic Effects
Titanium oxide nanoparticles	biomedical implants in bone [98]	impairments in the fetal brain development, dysregulated neurotransmitters, disturbed distribution of trace elements, synaptic plasticity, and disrupted signaling pathways [87]
	drug delivery, photodynamic therapy, cell imaging, biosensors and genetic engineering [12]	
		oxidative stress, inflammatory responses, apoptosis, genotoxicity, and impairment of cell components [88]
Iron oxide nanoparticles	magnetic particle imaging [99,100]	affected synaptic transmissions and nerve conduction, neural inflammation, apoptosis, induced neural antioxidant responses, and immune cell infiltration [90,101]
	cancer therapy [102]	
Silver nanoparticles	wound dressings and tissue scaffolds with antimicrobial activity, drug delivery systems [103]	oxidative stress, mitochondrial damage, and an increase in the calcium levels related to transporter/receptor mechanisms [92]
Gold nanoparticles	photodynamic therapy, photothermal therapy, x-ray imaging, and drug delivery systems [104]	astrogliosis, increased seizure activity, and cognition defects, such as attention, memory, and judgement impairments [93]
Silica nanoparticles	positron emission tomography and ultrasound imaging, protein and gene delivery, cancer therapy, and neurodegenerative diseases treatment [105]	oxidative stress and microglial function alterations [94]
		cognitive dysfunction and impairment and pathologies similar to neurodegeneration and synaptic changes [95]
Carbon-based nanoparticles	drug and gene delivery, cancer therapy, and fluorescence, photoacoustic and Raman imaging [106]	inflammatory response by activating microglial cells [97]
